# Increased CTLA-4^+^ T cells and an increased ratio of monocytes with loss of class II (CD14^+^ HLA-DR^lo/neg^) found in aggressive pediatric sarcoma patients

**DOI:** 10.1186/s40425-015-0082-0

**Published:** 2015-08-18

**Authors:** Pooja Hingorani, Mary L. Maas, Michael P. Gustafson, Paul Dickman, Roberta H. Adams, Masayo Watanabe, Francis Eshun, James Williams, Matthew J. Seidel, Allan B. Dietz

**Affiliations:** Center for Cancer and Blood Disorders, Phoenix Children’s Hospital, Phoenix, AZ USA; Human Cellular Therapy Lab, Department of Laboratory Medicine and Pathology, Mayo Clinic, 200 First Street SW, Rochester, MN 55905 USA; Department of Pathology, Phoenix Children’s Hospital, Phoenix, AZ USA; Orthopedic Oncology, Scottsdale, AZ USA; Division of Experimental Pathology, Department of Laboratory Medicine and Pathology, and Division of Immunology, Mayo Clinic, Rochester, MN USA

**Keywords:** Osteosarcoma, Ewing sarcoma, Immune phenotype, Immune suppression, Monocytes, Lymphocytes

## Abstract

**Background:**

There is little information regarding the composition of peripheral blood immunity in sarcoma patients and even less in the context of pediatric sarcomas. We describe the immune status using flow cytometry of peripheral blood in patients with osteosarcoma and Ewing sarcoma and demonstrate excessive CD14 in tumor tissues.

**Methods:**

Peripheral blood from patients with OS and ES was collected at diagnosis or relapse, and used for immune phenotyping of 74 different leukocyte phenotypes. Blood from young adult healthy volunteers was collected as controls. Tumor tissues were analyzed by immunohistochemistry.

**Results:**

Nineteen patients (average age = 14 y) and 16 controls (average age = 25y) were enrolled on study. Of the 74 phenotypes, 14 were different between sarcoma patients and HV. Sarcoma patients’ leukocytes contained a higher percentage of granulocytes (67 % sarcoma vs. 58 % HV; *p* = 0.003) and fewer lymphocytes (20 % sarcoma vs. 27 % HV; *p* = 0.001). Increased expression of CTLA-4 was seen in both T cells in sarcoma patients as compared to HV (*p* = 0.05). Increased CD14^+^ HLA-DR^lo/neg^ immunosuppressive monocytes were seen in sarcoma patients (*p* = 0.03); primarily seen in OS. Increased tumor necrosis factor receptor II expression was seen on CD14^+^ cells derived from sarcoma patients as compared to HV (*p* = 0.01). Massive infiltration of CD14^+^ cells was seen in OS (>50 % of cells in the majority of tumors) compared to ES (<10-25 % of cells). In contrast, both OS and ES had limited T cell infiltration (generally <10 % of cells).

**Conclusions:**

Pediatric sarcoma patients exhibit several immune phenotypic differences that were exacerbated in more severe disease. These phenotypes have the potential to contribute to immune suppression and may indicate potential targets for immune therapies.

**Electronic supplementary material:**

The online version of this article (doi:10.1186/s40425-015-0082-0) contains supplementary material, which is available to authorized users.

## Background

Pediatric bone sarcomas are rare tumors with overall incidence of 700 cases of osteosarcoma (OS) and 200 cases of Ewing sarcoma (ES) annually in the United States [1, 2]. Overall prognosis of advanced and relapsed pediatric sarcomas is dismal with 5-year event free survival less than 20 %. Treatment options for such patients have included chemotherapy, surgery and radiation therapy at the sites of recurrence although none of these approaches are generally curative. New approaches to therapy are needed to improve overall survival in this group of patients.

Immune dysregulation is an important contributing mechanism to the development of a variety of human cancers. Indeed, patients with common variable immunodeficiency are known to be at increased risk of developing lymphomas [3]. For example, EBV-associated solid tumors have been seen with increased frequency in patients with immune dysregulation such as after transplantation or in the setting of HIV/AIDS [4]. Immune system abnormalities have also been reported in solid tumors. Loss of CD27^+^ memory B cells, poor chemokine mediated trafficking of effector cells and inhibition of T-cell function by negative regulatory pathways have been noted in melanomas [5, 6]. Similarly, stage III and IV head and neck cancer patients have been shown to have down regulated NK cell and NK-T cell populations and up regulation of suppressor regulatory T cells (Tregs) [7]. These mechanisms together and alone are thought to represent the mechanism of tumor cell evasion from host’s immune surveillance.

Several reports exist of various immune abnormalities in patients with sarcomas including reduced Class I and Class II human leukocyte antigen (HLA) cell surface expression especially in metastatic and progressive tumors [8] and increased proportion of Tregs seen in bone marrow of patients with metastatic ES [9]. To date, analysis of most tumor immunity and particularly sarcomas have focused on single phenotypes, with extrapolation that these single phenotypes are representative of the immune system.

The lack of therapeutic options in sarcomas has led to interest in immune based therapies. For example, studies have used dendritic cells pulsed with tumor lysates or tumor specific antigens to stimulate anti-tumor immunity in ES and rhabdomyosarcoma [10–14]. Although safe, the success rate of therapy with dendritic cell vaccines in pediatric solid tumor patients has been modest. Improved responses for this and other immunotherapy approaches could benefit from a more complete understanding of the immune status of patients. In this study we have taken a broad approach to understanding immune dysfunction by assessing over 70 distinct immune phenotypes by flow cytometry. The compilation of phenotypes was used to generate an immune profile for each individual. The immune profiles of OS and ES patients and compared it to non-cancerous healthy volunteers in an effort to define the extent of immune dysfunction in pediatric sarcoma patients.

## Methods

### Patients

Institutional review board approval was obtained from Phoenix Children’s Hospital and the Mayo Clinic prior to the commencement of the study. Patients with newly diagnosed or relapsed OS and ES at Phoenix Children’s Hospital (PCH) were enrolled in this study after informed consent. HV between the ages of 18–30 years were provided with a screening questionnaire to exclude any underlying acute or chronic infections/ immunologic disorders as well as prior history of cancer. Up to 45 ml of blood in sodium heparin and 5 ml of blood in ethylenediamine tetraacetate (EDTA) was collected from patients and 10 ml of blood was collected from HV. Patients were required to have been off any myelosuppressive chemotherapy for four weeks prior to the time of sample collection. Blood was shipped overnight at room temperature to Mayo Clinic, Rochester, MN where it was processed for immune phenotyping.

### Immune-phenotyping peripheral blood from healthy volunteers and sarcoma patients

Leukocytes were analyzed by direct antibody staining of whole blood and analyzed by flow cytometry. The protocol for whole blood staining was adapted from Appay et al. with full details described in Gustafson et al. [15, 16]. Data were acquired on a BD FACSCalibur flow cytometer (Becton Dickinson, Frankin Lakes, NJ) that was calibrated on the day of use and analyzed with FlowJo (Tree Star Inc., Ashland, OR) software. Cell counts were measured using Trucount™ tubes according to manufacturer’s directions (BD Biosciences, San Jose, CA). Data were analyzed with Cell Quest and Multiset (Becton Dickinson) software. The antibodies against cell surface markers used for these studies were obtained from BD Biosciences. The specific markers are listed in Additional file 1. Examples from HV and sarcoma patients for CD14, HLA-DR, CD4 and CTLA-4 can be found in Additional file 2. Gating strategies and descriptions of other immune phenotypes have been described elsewhere [16–20].

### Patient tissue samples and immunohistochemistry

Formalin-fixed paraffin embedded archived diagnostic biopsy samples of the patients enrolled in this study were retrieved from the PCH tissue bank. Serial 5 μm sections were obtained for immunohistochemical analysis. Slides were loaded onto Bond-III auto-stainer, deparaffinized and retrieved in EDTA for 30 min. Bond polymer refined red detection was used for the CD3 antibody. Primary CD3 antibody (DAKO #M-7254 Mouse Monoclonal (clone: F7.2.38)) was incubated for 15 min followed by post primary-alkaline phosphatase (AP) for 30 min, polymer- AP for 30 min and red chromagen for 15 min. Slides were rinsed in Bond Wash between each of the steps. Slides were counterstained with hematoxylin and taken off the auto-stainer, dehydrated in graded ethyl alcohol, cleared in xylene and cover slipped in permanent mounting media. CD14 staining (Sigma #HPA001887 Rabbit Polyclonal) was as above except the EDTA retrieval was for 20 min and brown chromagen was used. The slides were reviewed by two independent pathologists and graded for monocytic (CD14) and lymphocytic (CD3) infiltration as follows: 1+ (<10 %), 2+ (10-25 %), 3+ (25-50 %) and 4+ (>50 %).

### Multiparameter analysis and hierarchical clustering

Hierarchical clustering of immune markers was performed as described by Gustafson et al. [18]. Briefly, cell counts from 9 immune markers (Granulocytes, lymphocytes, monocytes, T cells, B cells, NK cells, CD4^+^ T cells, regulatory T cells, and CD14^+^HLA-DR^lo/neg^ monocytes) were either measured directly in cells/μl or converted into cells/μl. An immune marker ratio for each individual (HV and patients) was determined by dividing the individual immune marker value by the mean value of the entire healthy volunteer cohort. The marker ratios for each volunteer and patient were imported into Partek Genomics Suite 6.6 software (Partek Inc., St. Louis, MO) and log-transformed for hierarchical clustering. Hierarchical clustering was performed by unsupervised agglomerative Euclidean average linkage clustering. Groups were classified as having ≥5 individuals.

### Statistical analysis

All graphical representations and data analysis were performed using Prism 5.0 software (GraphPad, La Jolla, CA). For the immune phenotyping results, values between groups of data were tested for statistical significance using the Mann Whitney *t* test for unmatched groups and the non-parametric Spearman test for correlations. The Fisher’s exact test was used to determine the significance of the distribution of sarcoma and HV immune profiles. The significance level was set at probability of significance set at less than 0.05 with specific calculated *P* values provided when applicable. The data for the tissue IHC is reported descriptively due to small patient numbers.

## Results

### Patient characteristics

Twenty patients, 11 with OS and 9 with ES were enrolled on the study along with 16 HV. One patient with ES was ineligible for immmunephenotyping analysis due to inadequate amount of blood collected for analysis. Median age of the patients was 14 years (range 6–22 years); and of the HV was 25 years (range 20–30 years). Patient demographics, diagnosis, and clinical data are listed in Table 1.

**Table 1:** Patient characteristics

Patient number	Diagnosis	Age (years)	Gender	Primary site	Metastases	Enrollment	Prior therapy	From last therapy	Blood analysis	Tissue analysis	(months since)
1	OS	16	Male	Distal femur	B/L Pulmonary	ND	None	NA	Yes	Yes	Local Relapse 30 months, alive, NED
2	OS	18	Male	Pelvis	None	ND	None	NA	Yes	No	DOD
3	OS	12	Female	Proximal humerus	None	ND	None	NA	Yes	Yes	NED 24 mths
4	OS	10	Female	Proximal humerus	U/L pulmonary	ND	None	NA	Yes	Yes	Local Relapse 27 months, alive, NED
5	OS	21	Male	Distal femur	None	ND	None	NA	Yes	No	NED 27 mths
6	OS	13	Female	Proximal tibia	None	ND	None	NA	Yes	Yes	Died of pulmonary embolism during surgery
7	OS	16	Female	Distal femur	None	ND	None	NA	Yes	Yes	NED 20 mths
8	OS	14	Female	Proximal tibia	None	ND	None	NA	Yes	Yes	NED 20 mths
9	OS	7	Male	Distal femur	None	ND	None	NA	Yes	Yes	NED 19 mths
10	OS	15	Male	Craniofacial	None	ND	None	NA	Yes	No	NED 19 mths
11	OS	12	Female	Distal femur	B/L Pulmonary	ND	None	NA	Yes	Yes	DOD 22 mths
12	ES	14	Female	Scapula	None	Relapse	VDC/ IE	12 weeks	Yes	Yes	DOD 36 mths
13	ES	19	Male	Pelvis	B/L Pulmonary	Relapse	VDC/ IE	8 weeks	Yes	Yes	DOD 28 mths
14	ES	18	Male	Spine	None	ND	None	NA	Yes	No	Died in a car accident 6 mths after diagnosis
15	ES	13	Male	Pelvis	None	ND	None	NA	Yes	Yes	NED 29 mths
16	ES	22	Male	Chest wall	None	Relapse	VDC/ IE	4 years	Yes	No	NED 72 mths
					Diffuse bone and						DOD 13
17	ES	6	Female	Scapula	bone marrow	ND	None	NA	No	Yes	mths
18	ES	13	Female	Sacrum	None	ND	None	NA	Yes	Yes	NED 20 mths
19	ES	7	Male	Mandible proximal	None	ND	None	NA	Yes	No	NED 17 mths
20	ES	13	Male	Humerus	None	ND	None	NA	Yes	Yes	NED 15 mths

DOD- died of disease

NED- no evidence of disease

ND- new diagnosis

VDC/IE- Vincristine, doxorubicin, cyclophosphamide, ifosfamide and etoposide

### Pediatric sarcoma patients have an altered peripheral blood leukocyte distribution

**Fig. 1:**
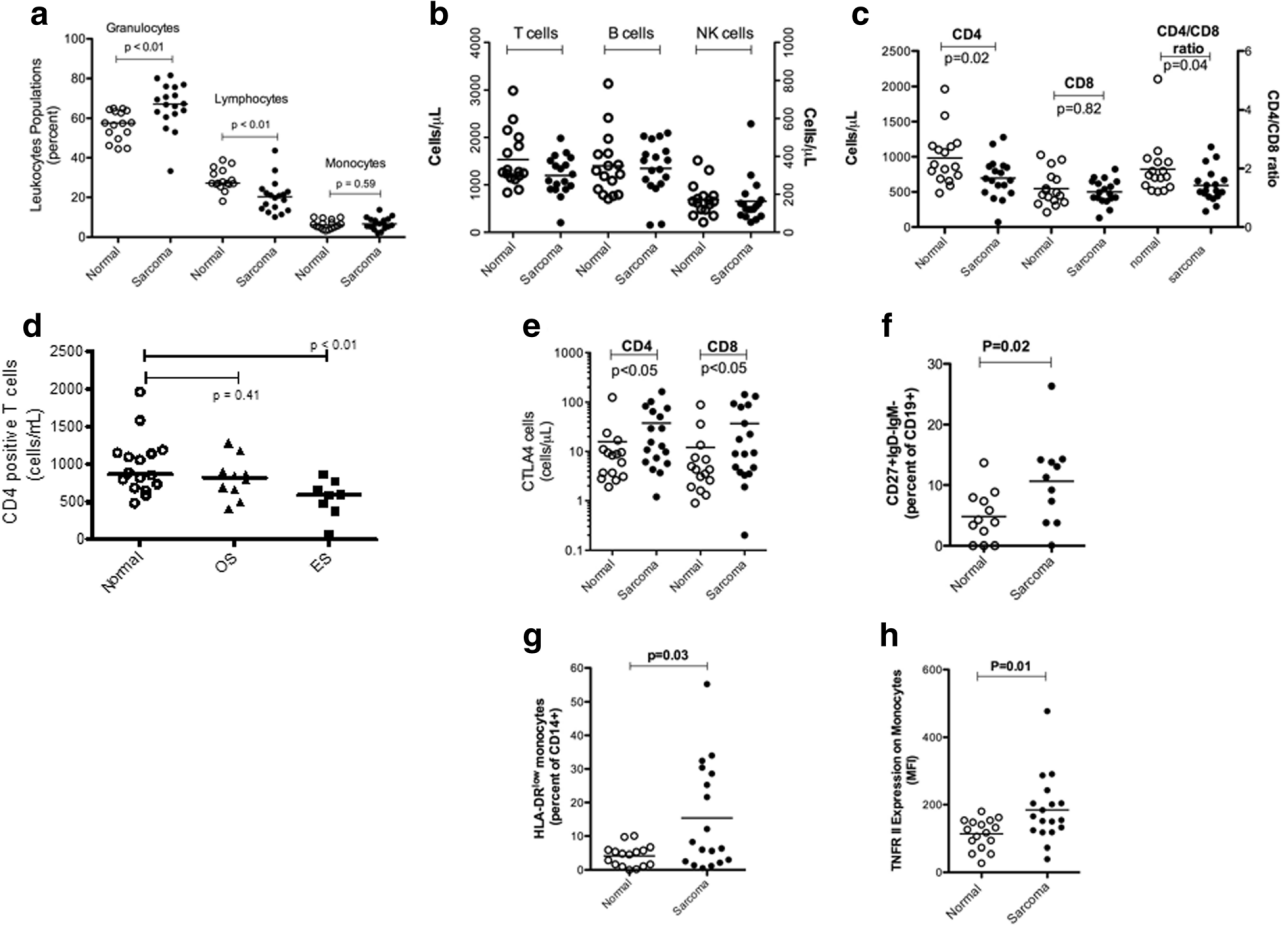
Alterations in peripheral blood immune phenotypes in pediatric sarcoma patients. Immune phenotypes from healthy volunteers and pediatric sarcoma patients were measured by flow cytometry. **a**. The percentages of granulocytes, lymphocytes, and monocytes of total leukocytes as measured by forward and side scatter properties in healthy volunteers and sarcoma patients. Comparison of cell counts (Cells/μl) of: **b**, T cells (Left axis), B cells (Right axis), and NK cells (Right axis); **c**, CD4, CD8 and the CD4:CD8 ratio (CD4 cell counts/CD8 cell counts); **d**, CD4 cell counts in osteogenic sarcoma versus Ewing’s sarcoma; **e**, CTLA-4^+^ CD4 and CD8 cells. Increases in the percentages of **f**, CD19^+^CD27^+^IgD^−^IgM^−^ B cells and **g**, CD14^+^HLA-DR^lo/neg^ monocytes; **h**, and an increase of expression of TNFRII (by mean flourescenc intensity) on monocytes was observed in sarcoma patients. P values are listed where values are < 0.05

In effort to understand the breadth and depth of immunological changes in sarcoma patients, we analyzed the basic white blood cell composition in the peripheral blood of sarcoma patients (*n* = 19) and HV (*n* = 16) using flow cytometry. Leukocytes from sarcoma patients had a higher percentage of granulocytes (67 % sarcoma patients vs. 58 % HV; *p* = 0.003) and a lower percentage of lymphocytes (20 % sarcoma patients vs. 27 % HV; *p* = 0.001). There was no difference in the percentage of monocytes between the two groups (Fig. 1a). No difference was seen in the total T-cell, B-cell and NK cell population between sarcoma patients and HV (Fig. 1b). However, on analysis of T-lymphocyte subsets the sarcoma patients had lower CD4 T cells as compared to HV (697 CD4 cells/ μL vs. 983 CD4 cells/ μL respectively; *p* = 0.02). No difference was seen in CD8 T cells between the 2 groups (*p* = 0.82) leading to an altered CD4/CD8 ratio in patients (*p* = 0.04) (Fig. 1c). This difference in CD4 T cells was primarily seen in ES patients (596 CD4 cells/ vs. 874 CD4 cells/μL; *p* = 0.01) (Fig. 1d).

### Pediatric sarcoma patients have evidence of immune modulating phenotypes

In addition to the leukocyte differences observed above, we identified several other altered phenotypes in sarcoma patients. Sarcoma patients had increased expression of CTLA-4, a T-cell inhibitory receptor, on both CD4 (38 % sarcoma vs. 16 % HV; *p* = 0.05) and CD8 T cells (37 % sarcoma vs. 12 % HV; *p* = 0.05) as compared to HV (Fig. 1e). In the B-cell compartment, an increase in class-switched memory B-cells (CD27 + IgM-IgD-) was seen in sarcoma patients vs. HV (115 vs. 5 % respectively; *p* = 0.02) (Fig. 1f). We also analyzed the peripheral blood of OS and ES patients for a previously described class of immune suppressive monocytes, CD14^+^HLA-DR^lo/neg^ monocytes [16, 20–22]. These cells have been shown in adult glioblastoma, lymphoma, prostate cancer and CLL to have effects both directly (with an inability to generate dendritic cells, and inhibit T cell proliferation) and systemically through expression of arginase one [16, 20–22]. Increased CD14^+^HLA-DR^lo/neg^ immunosuppressive monocytes were seen in sarcoma patients as compared to HV (15 % vs. 4 % respectively; *p* = 0.03) (Fig. 1g). This group effect existed because of the preponderance of these cells in OS patients (19 % vs. 4 %; *p* = 0.01) In addition, increased expression of tumor necrosis factor receptor II was seen on CD14^+^ monocytes derived from sarcoma patients as compared to HV (*p* = 0.01) (Fig. 1h). Notably, we did not see any increase in regulatory T cells or LIN^−^CD33^+^HLA-DR^−^ myeloid derived suppressor cells (MDSC) in peripheral blood of sarcoma patients as compared to HV. The complete results for all immunophenotypes in HV and sarcoma patients and associated P values are listed in Additional file 3.

### Hierarchical clustering of immune phenotypes reveals immunosuppressive profiles in sarcoma patients

**Fig. 2:**
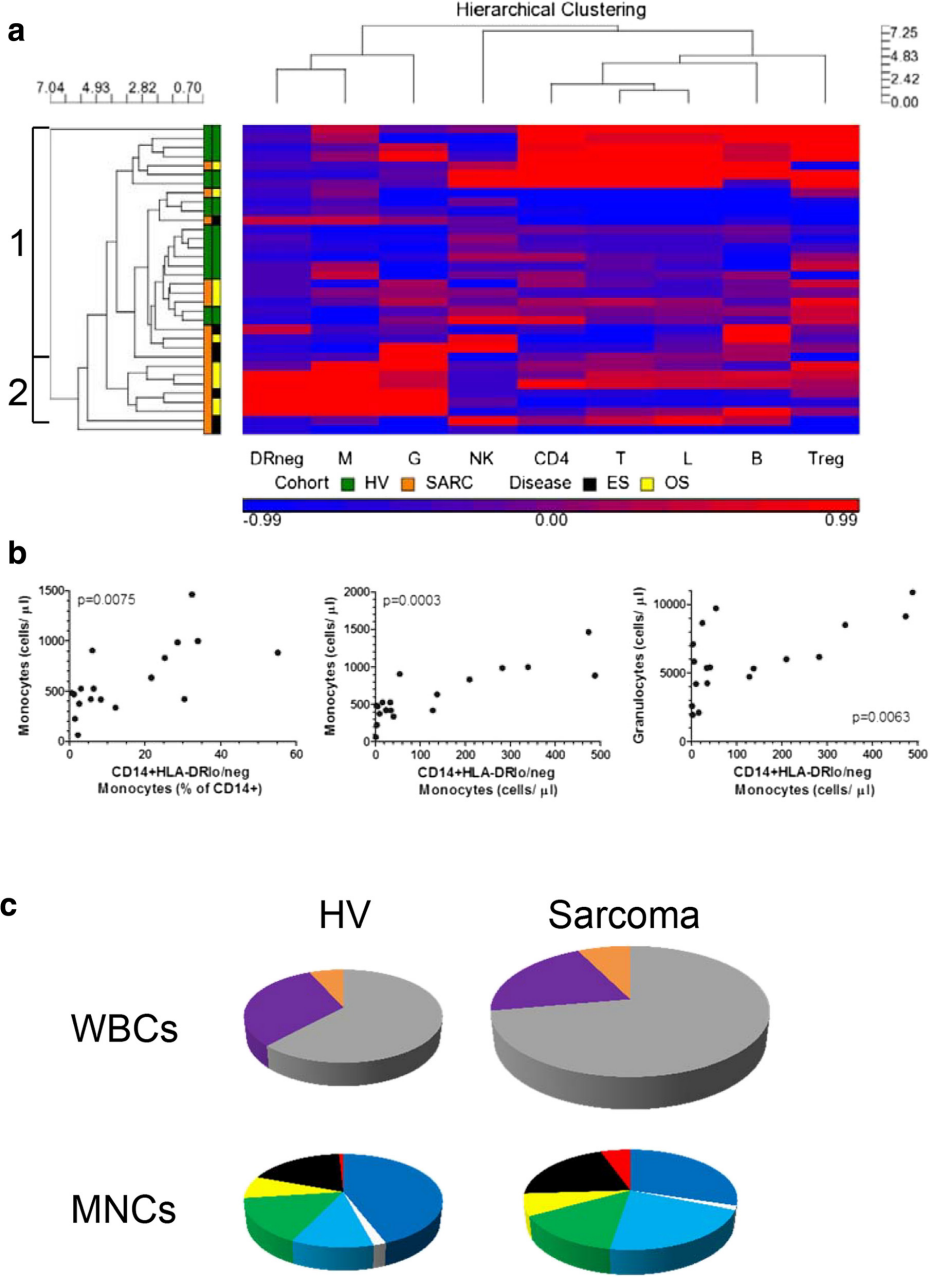
Hierarchical clustering of immune phenotypes reveals potential differential subgrouping of sarcoma patients based on compositional changes in peripheral blood immune phenotypes. **a**. Hierarchical clustering dendrogram showing the relationships of healthy volunteers and sarcoma patients based on nine immune phenotypes (DRneg- CD14^+^HLA-DR^lo/neg^ monocytes; M- monocytes; G- granulocytes; NK- natural killer cells; CD4- CD4 T cells; T- T cells; B- B cells; Treg- CD4^+^CD25^+^CD127^lo^ T cells). Profiles were defined by including a minimum of 5 individuals. Values shaded in red indicate values above the mean of the HV value and blue indicates values below. Two profiles of groups were identified. **b**. Hierarchical clustering also can reveal relationships of immune phenotypes to each other. The percentage and cell counts of CD14^+^HLA-DR^lo/neg^ monocytes correlated to the cell counts of monocytes and the cell counts of CD14^+^HLA-DR^lo/neg^ monocytes correlated to the cell counts of granulocytes. **c**. Illustrative comparisons of the compositional differences in healthy volunteers and sarcoma patients. For total white blood cells (WBCs); the percentages of granulocytes (gray), lymphocytes (purple), and monocytes (orange) are shown. The pie graphs are also sized based on cell counts where the sarcoma pie graph is sized in proportion to the HV pie graph. Mononuclear cells (MNCs) show CD4 T cells (dark blue), Tregs (white), CD8 T cells (light blue), B cells (green), NK cells (yellow), CD14^+^HLA-DR^+^ monocytes (black), and CD14^+^HLA-DR^lo/neg^ monocytes (red). The sarcoma pie graph is sized in proportion to the HV graph

The generation of immune profiles from the measurements of multiple immune phenotypes provides a more complete picture of the immune status of patients. We have previously shown that patients with profiles other than those seen in healthy patients experience a negative impact on cancer patient survival [18]. We applied this approach to our sarcoma cohort using nine immune phenotypes that have been previously shown to cluster patients in clinically meaningful groups [18]. Clustering analysis of HV and sarcoma patients revealed two major groups (Fig. 2a). Group 1 contained 16 HV and 10 sarcoma patients whereas group 2 had 6 patients with no HV (p = 0.0177, Fisher’s exact test). 1 HV and 2 sarcoma patients did not cluster into any group. ES and OS patients did not differentially subgroup in this analysis. This result suggests that sarcoma patients in Group 1 have an immune profile similar to healthy volunteers and that sarcoma patients in Group 2 have an abnormal immune profile. While no patients in group 1 died due to disease progression, we found that 3 out of 8 patients that clustered outside of group 1 died from their disease. These results are similar to our findings observed in a larger cohort of adult cancer patients where the abnormal immune profiles adversely affected survival [18]. One of the distinguishing immunophenotypes between sarcoma patients in group 1 versus 2 was the levels of CD14^+^HLA-DR^lo/neg^ monocytes. Sarcoma patients in profile 1 had CD14^+^HLA-DR^lo/neg^ monocytes similar to the levels in healthy volunteers in terms of cell counts and percentages (mean: 39.9 cells/μl and 9.2 ± 9.6 % of CD14^+^ monocytes) whereas sarcoma patients in group 2 had significantly elevated CD14^+^HLA-DR^lo/neg^ monocytes (307.7 cells/μl (*p* = 0.0087 vs. profile 1 and 30.2 ± 15.9 % (*p* = 0.011)). Hierarchical clustering can also reveal novel relationships between immune phenotypes. Granulocytes, monocytes, and CD14^+^HLA-DR^lo/neg^ monocytes clustered together suggesting that changes in these populations are related. Indeed, the levels of CD14^+^HLA-DR^lo/neg^ monocytes from all sarcoma patients, in both cell counts (cells/μl) and percentage of total CD14^+^ monocytes, were positively correlated to the monocyte cell counts (Fig. 2b). In addition, CD14^+^HLA-DR^lo/neg^ monocyte cell counts correlated to the granulocyte counts. Finally, we can quantify the entire leukocyte population of sarcoma patients by representing the immune profiles as a pie chart. Figure 2c shows pie charts that represent an immune profile from healthy volunteers and sarcoma patients in both terms of total leukocytes (WBCs) and mononuclear cells (MNCs). The size of the pie chart is relative to the healthy volunteer profile. The total leukocyte population of sarcoma patients is approximately 50 % larger than the healthy volunteer profile whereas the mononuclear compartment is very similar. Although the total size of the sarcoma MNC compartment is similar to healthy volunteers, the distribution of subsets is quite different. Taken together, these data identify an immune profile in sarcoma patients characterized by increased CD14^+^HLA-DR^lo/neg^ monocytes, total monocytes, and granulocytes.

### Significant infiltration of CD14^+^ cells is present in osteosarcoma primary tumors

**Fig. 3:**
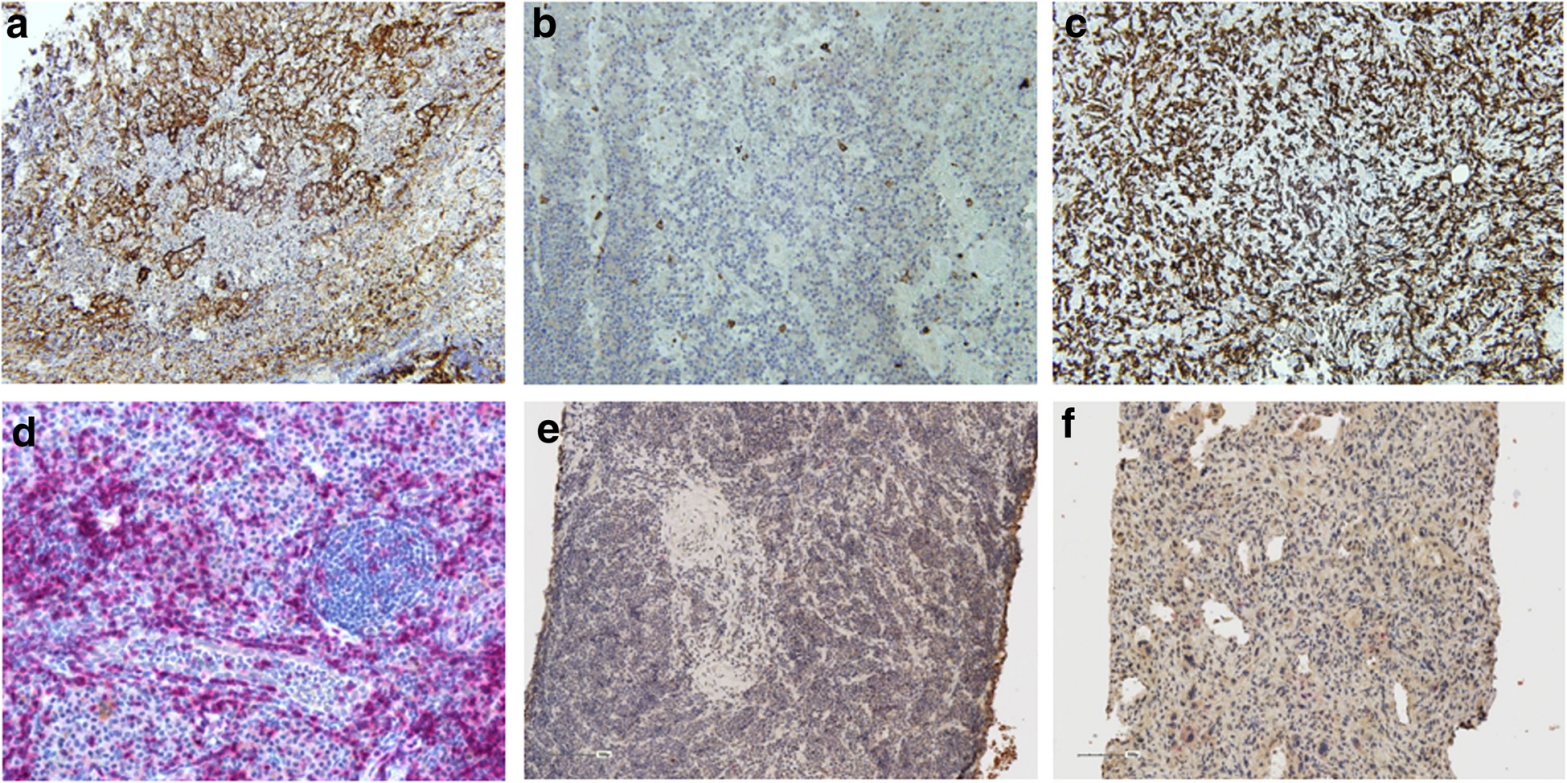
CD14^+^ monocytes accumulate in sarcoma tumors. Immunohistochemical analysis was performed using CD14 antibody for monocytic infiltration and CD3 antibody for lymphocytic infiltration on primary tumor tissues of OS and ES patients; **a**, **b**, **c** represent CD14 staining (brown) in tonsil control, ES and OS respectively; **d**, **e**, **f** represent CD3 staining (red) in tonsil control, ES and OS respectively

We have previously shown that for glioblastoma and renal cell carcinoma patients, an increase in peripheral blood CD14^+^HLA-DR^lo/neg^ monocytes was associated with increased infiltration of CD14^+^ cells within the tumor [16, 23]. We analyzed the diagnostic biopsy samples of patients enrolled on this study for lymphocytic and CD14^+^ infiltration by immunohistochemistry. Eight OS patient samples and six ES samples were available for analysis. CD3 cell surface marker was used for lymphocyte identification in addition to CD14 identification in tumor tissues. Minimal tumor-associated T cell infiltration was seen in both OS and ES tumors (<10 % in 12/14 tumors and 10-25 % in 2/14 tumors). In contrast, OS specimens had marked CD14^+^ infiltration in the majority of the tissues (>50 % in 7/8; 10-25 % in 1/8 tumors) (Fig. 3).

## Discussion

Evidence of immune dysregulation is found in several human cancers and is believed to be an important escape mechanism for the tumor cells from the host immune system. Limited data exists in pediatric bone sarcomas regarding the underlying immune deficits. In the current study, we performed detailed immune phenotyping analysis in patients with OS and ES to better characterize the immune system of these patients.

Our data demonstrates that pediatric sarcoma patients exhibit alterations in the distribution leukocytes whereby granulocyte cell counts are elevated in peripheral blood thus reducing the overall percentage of lymphocytes and CD4 lymphopenia that is more profound in ES patients. We acknowledge that the healthy volunteer cohort was older than the pediatric sarcoma cohort; however, there are several reports in the pediatric literature that suggest that the frequencies and cell counts between the ages of 6–24 do not substantially change [24–26]. Reference values for many of the phenotypes we measured here have not been established or reported. Therefore, our data provides a framework for future studies in pediatric sarcoma. Lymphopenia at diagnosis in adults is considered to be a poor prognostic factor in soft tissue sarcoma [27]. In both OS and ES, early lymphocyte recovery after chemotherapy courses has been shown to be a prognostic indicator for improved overall survival [28, 29]. In preclinical studies, immune reconstitution prevented the development of metastasis in a murine model of OS [30]. Our data goes beyond the description of abnormal leukocyte distributions; that is, we have identified specific alterations in immune populations that have previously been implicated in immune suppression in a variety of cancers, including B-cell non-Hodgkin lymphoma, prostate, CLL and GBM [16, 20–22].

Differences in immune markers were observed both in peripheral blood and in tumor tissues of OS and ES patients. ES patients demonstrated a lower absolute CD4 T cell count as compared to HV but no such difference was observed in OS. We did not observe a difference in the absolute Treg count in peripheral blood of ES patients, which is contrary to the data published before showing higher Tregs in the bone marrow of ES patients with metastatic disease. Increased expression of the inhibitory T cell receptor CTLA-4 on CD4 and CD8 T cells in sarcoma patients may have direct implications for immune therapy. CTLA-4 expression was noted in OS cell lines and patient tumor samples [31] and CTLA-4 + 49G/A polymorphism was associated with increased risk of developing OS [32]. Combined treatment with anti-CTLA-4 antibody and tumor lysate-pulsed dendritic cells in mice with OS lead to decreased Tregs and increased CD8 T cells in metastatic tumor leading to decreased metastasis and increased EFS [33]. Taken together, CTL-4 targeted therapy may be beneficial, and it will be important to evaluate the effect of this therapy based on the expression on T cells or tumor tissue. Not all of our data can be interpreted in the context of immune suppression. For example, CD69 (typically a marker of T cell activation) was seen on CD4^+^ T cells. Additional markers of T cell anergy and/or exhaustion (i.e. PD-1 and TIM-3) and functional data may help clarify the impact of these alterations in sarcoma patients.

Increased numbers of CD14^+^ HLA-DR^lo/neg^ monocytes in the peripheral blood of OS patients has previously been shown to be immunosuppressive in a variety of cancers such as prostate cancer, non-Hodgkin lymphoma (NHL), glioblastoma multiforme, and chronic lymphocytic leukemia [16, 20–22]. CD14^+^ HLA-DR^lo/neg^ monocytes suppress autologous T cell proliferation and are defective in their ability to differentiate into mature dendritic cells in cancer patients [34]. In patients with NHL, elevated levels of CD14^+^ HLA-DR^lo/neg^ monocytes were associated with more aggressive disease and suppressed immune functions via multiple mechanisms including altering STAT signaling, increasing IDO, arginase, iNOS, NOX2 and VEGF expression, preventing DC maturation, altering co-stimulatory and cytokine expression and decreasing antigen uptake as summarized in a recent review by Laborde et al. [34]. In addition, others have shown that CD14^+^ HLA-DR^lo/neg^ monocytes suppress T cell function and proliferation by secreting arginase I, IDO, and TGF-beta [35–37]. As the interaction between tumor cells and monocytes appears to promote the loss of HLA-DR on monocytes [23, 16], we hypothesized that CD14^+^ monocytes would likely be present in the tumor microenvironment. In primary tumor tissues, both OS and ES tissues had limited tumor-infiltrating lymphocytes but 87 % of OS tissues (*n* = 8) had a significant (>50 %) CD14+ cell infiltration while 83 % of ES tissues (*n* = 6) had less than 25 % CD14^+^ cell infiltration. We believe that the association of greater tumor infiltration of CD14^+^ cells and greater numbers of CD14^+^ HLA-DR^neg^ cells with advanced disease strongly suggests that these cells may be part of the pathogenesis of these tumors and should be further investigated as high priority targets for new therapies.

## Conclusions

This study was designed to establish a global overview of immune status in this rare patient cohort and to identify immune phenotypes that are relevant to the pathology of pediatric sarcomas. We have identified several immune phenotypes that are altered in pediatric sarcomas including elevated CD14^+^ HLA-DR^lo/neg^ cells, elevated CTLA-4^+^ T cells, and decreased CD4 T cells. This initial exploration of immune phenotypes will likely need to be validated in a larger cohort with fewer, more targeted phenotypes. Additionally, future studies should be followed by functional studies to further develop immunotherapy treatment protocols focusing on correcting specific immune deficiencies. Specific targets of immunotherapy in OS and ES will likely include reconstitution of the CD4 T cell pool, removing inherent immune suppression (such as reversing the CTLA-4 phenotypes), reducing granulocytes and eliminating CD14^+^ HLA-DR^lo/neg^ cells. Recent trials applying immune inhibitory pathway inhibitors such as anti-CTLA-4 [38] and anti-PD-1 [39] agents in metastatic melanoma demonstrated a significantly improved progression free survival in patients and are a testament to the importance of immune inhibitory pathways in cancer pathogenesis. It is our belief that similar therapeutic strategies may be effective in pediatric sarcomas. These studies in combination with extensive immune characterization may lead to improvements in matching appropriate therapies to patients, improving appropriate treatment time and identifying the correct immune modulating therapy combinations for patients.

## Additional files

Additional file 1:
**Antibodies used for flow cytometry along with their corresponding cell phenotypes.** (DOCX 17 kb) Additional file 2:
**Gating strategies for selected phenotypes.** Dot plots and/or histograms showing the gating for CD14^+^HLA-DR^lo/neg^ monocytes (healthy volunteer and sarcoma patient; Top two rows) and CTLA-4 (bottom two rows). (TIFF 219 kb) Additional file 3:
**Immune phenotypes of HV and sarcoma patients.** Table of phenoyptes shows mean and standard deviation of healthy volunteers (HV), pooled sarcoma patients, and patients subgrouped by disease (osteogenic vs. Ewings’ sarcoma). P values are also listed among the comparisons. (DOCX 40 kb)

## Abbreviations

AP: Alkaline phosphatase
CTLA-4: Cytotoxic lymphocyte antigen 4
EBV: Epstein-Barr virus
EDTA: Ethylenediaminetetraacetic acid
ES: Ewings’ Sarcoma
HLA: Human leukocyte antigen
HV: Healthy volunteers
OS: Osteosarcoma
Tregs: Regulatory T cells
Y: Years
