# Supplementary material for: Increased CTLA-4+ T cells and an increased ratio of monocytes with loss of class II (CD14+ HLA-DRlo/neg) found in aggressive pediatric sarcoma patients
Source: J Immunother Cancer. 2015 Aug 18;3:35. doi: 10.1186/s40425-015-0082-0 (PMC4539889; doi:10.1186/s40425-015-0082-0)
Supplement: Additional file 1: — Antibodies used for flow cytometry along with their corresponding cell phenotypes. (DOCX 17 kb) [file 40425_2015_82_MOESM1_ESM.docx]

| **Additional File 1:** Antibodies used for flow cytometry along with their corresponding cell phenotypes | | | | |
| --- | --- | --- | --- | --- |
|  | | | | |
| **FITC** | **PE** | **PerCp** | **APC** | **Phenotype** |
| CD4 | CD8 | CD3 | HLA-DR | T cell subsets |
| CCR7 | CD45RO | CD4 | CD62L | Central/ effector memory T cells |
| CD4 | CD8 | CD28 | CTLA4 (CD152) | Co-stimulatory signals |
| CCR7 | CD45RO | CD8 | CD62L | Central/ effector memory T cells |
| CD80 | CD86 | HLA-DR | CD14 | Co-stimulatory signals |
| Lineage 1 | B7-H1 (CD274) | HLA-DR | CD33 | Conventional MDSCs |
| CD206 | CD163 | HLA-DR | CD14 | Alternatively activated monocytes |
| CD64 | CD16 | HLA-DR | CD14 | M1 and M2 monocytes |
| TNFRI | TNFRII | HLA-DR | CD14 | Signaling molecules on monocytes |
| TLR4 | IL-4Rα (CD124) | HLA-DR | CD14 | Signaling molecules on monocytes |
| TLR9 | M-CSFR (CD115) | HLA-DR | CD14 | Signaling molecules on monocytes |
| CD64 | CD13 | CD15 | CD14 | Neutrophils/ monocytes |

| TruCount Tubes | | | | |
| --- | --- | --- | --- | --- |
| CD3/CD16+CD56/CD45/CD19 | | | | Absolute T cell, B cell, NK cell per microliter |
| CD45 | CD127 | CD4 | CD25 | Absolute CD4, Tregs per microliter |

FITC: Fluorescein Isothiocyanate; PE: Phycoerythrin; PerCp: Peridinin chlorophyll protein complex; APC: Allophycocyanin
