# Supplementary figures and images for: Increased CTLA-4+ T cells and an increased ratio of monocytes with loss of class II (CD14+ HLA-DRlo/neg) found in aggressive pediatric sarcoma patients
Source: J Immunother Cancer. 2015 Aug 18;3:35. doi: 10.1186/s40425-015-0082-0 (PMC4539889; doi:10.1186/s40425-015-0082-0)

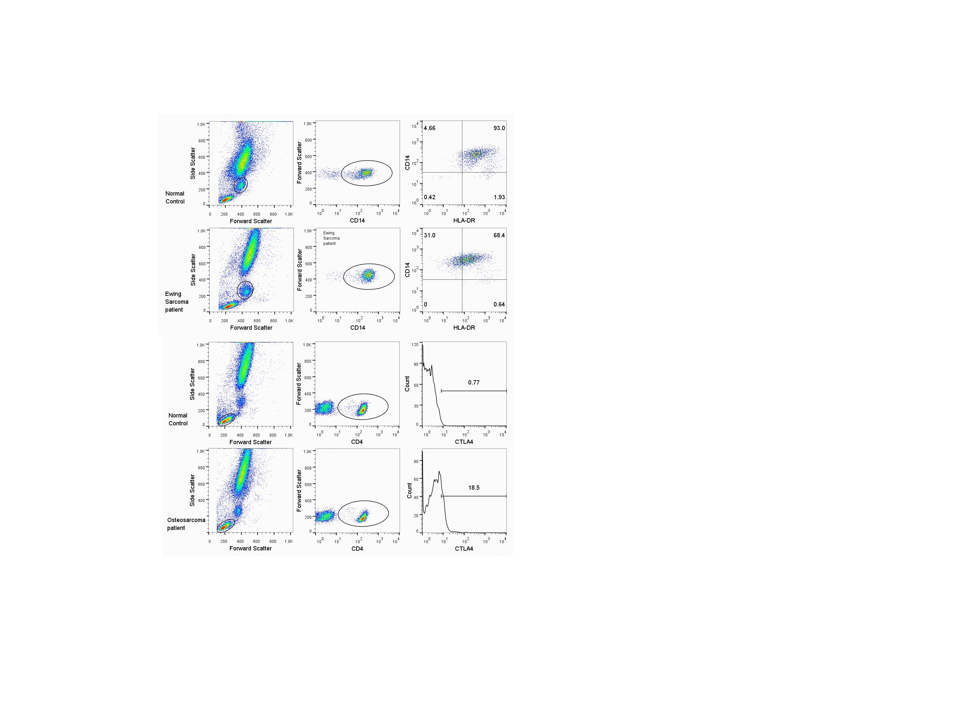

Supplement: Additional file 2: — Gating strategies for selected phenotypes. Dot plots and/or histograms showing the gating for CD14+HLA-DRlo/neg monocytes (healthy volunteer and sarcoma patient; Top two rows) and CTLA-4 (bottom two rows). (TIFF 219 kb) [file 40425_2015_82_MOESM2_ESM.tif]
