# Supplementary material for: Increased CTLA-4+ T cells and an increased ratio of monocytes with loss of class II (CD14+ HLA-DRlo/neg) found in aggressive pediatric sarcoma patients
Source: J Immunother Cancer. 2015 Aug 18;3:35. doi: 10.1186/s40425-015-0082-0 (PMC4539889; doi:10.1186/s40425-015-0082-0)
Supplement: Additional file 3: — Immune phenotypes of HV and sarcoma patients. Table of phenoyptes shows mean and standard deviation of healthy volunteers (HV), pooled sarcoma patients, and patients subgrouped by disease (osteogenic vs. Ewings’ sarcoma). P values are also listed among the comparisons. (DOCX 40 kb) [file 40425_2015_82_MOESM3_ESM.docx]

| **Additional File 3:** Immune phenotypes of HV and sarcoma patients | | | | | | | | | |
| --- | --- | --- | --- | --- | --- | --- | --- | --- | --- |
|  | | | | | | | | | |
|  |  |  |  |  |  | p value | | | |
|  | **HV** | **pooled** | **OS** | **ES** |  | **pool vs HV** | **OS vs HV** | **ES vs HV** | **OS vs ES** |
| **Age** | 25.13 | 14.78 | 15.00 | 16.50 |  | < 0.01 | <0.01 | <0.01 | 0.51 |
|  | 2.92 | 3.80 | 3.28 | 3.73 |  |  |  |  |  |
| **Granulocytes (percent of leukocytes)** | 56.53 | 66.30 | 65.38 | 67.46 |  | <0.01 | 0.02 | 0.01 | 0.36 |
|  | 7.36 | 11.48 | 7.96 | 15.36 |  |  |  |  |  |
| **Granulocytes cells/µL** | 4027 | 5991 | 6353 | 5375 |  | 0.01 | <0.01 | 0.21 | 0.31 |
|  | 1277 | 2590 | 2918 | 2530 |  |  |  |  |  |
| **Lymphocytes (percent of leukocytes)** | 28.84 | 20.46 | 24.85 | 20.34 |  | <0.01 | 0.01 | <0.01 | 0.50 |
|  | 5.36 | 8.29 | 17.09 | 10.33 |  |  |  |  |  |
| **Lymphocytes cells/µL** | 2058 | 1699 | 1837 | 1528 |  | 0.31 | 0.73 | 0.17 | 0.27 |
|  | 754.2 | 551.3 | 471.1 | 626.3 |  |  |  |  |  |
| **Monocytes (percent of leukocytes)** | 6.30 | 6.81 | 7.77 | 5.61 |  | 0.59 | 0.18 | 0.55 | 0.20 |
|  | 2.25 | 2.99 | 2.96 | 2.74 |  |  |  |  |  |
| **Monocytes cells/µL** | 431.0 | 608.8 | 701.8 | 452.2 |  | 0.16 | 0.09 | 0.90 | 0.27 |
|  | 142.5 | 339.9 | 379.5 | 278.7 |  |  |  |  |  |
| **T cell subsets** |  |  |  |  |  |  |  |  |  |
| **CD3^+^ cells/µL** | 1535.00 | 1199.00 | 1375.00 | 979.60 |  | 0.15 | 0.76 | 0.03 | 0.04 |
|  | 591.20 | 416.20 | 337.10 | 419.10 |  |  |  |  |  |
| **CD4^+^ cells/µL** | 983.00 | 697.60 | 812.60 | 553.90 |  | 0.04 | 0.41 | <0.01 | 0.05 |
|  | 381.90 | 286.60 | 272.10 | 248.60 |  |  |  |  |  |
| **CD4+ (percent of CD3+ lymphocytes)** | 62.59 | 53.25 | 56.00 | 49.81 |  | <0.01 | 0.23 | <0.01 | 0.17 |
|  | 4.86 | 11.78 | 10.83 | 12.72 |  |  |  |  |  |
| **CD8^+^ cells/µL** | 547.70 | 501.40 | 561.90 | 425.70 |  | 0.82 | 0.58 | 0.28 | 0.12 |
|  | 243.70 | 174.70 | 142.70 | 190.30 |  |  |  |  |  |
| **CD8+ (percent of CD3+ lymphocytes)** | 33.17 | 40.25 | 38.11 | 42.92 |  | 0.03 | 0.22 | 0.01 | 0.62 |
|  | 4.44 | 11.45 | 9.94 | 13.30 |  |  |  |  |  |
| **CD4/CD8 ratio** | 1.976 | 1.422 |  |  |  | 0.04 |  |  |  |
|  | 0.9191 | 0.5739 |  |  |  |  |  |  |  |
| **Regulatory CD4+25+127^low^ cells/µL** | 39.19 | 30.42 | 36.11 | 23.07 |  | 0.11 | 0.68 | 0.02 | <0.01 |
|  | 12.96 | 10.92 | 6.35 | 11.99 |  |  |  |  |  |
| **Regulatory CD4+25+127^low^ (percent of CD4+)** | 7.15 | 8.20 | 8.24 | 8.15 |  | 0.23 | 0.28 | 0.42 | 0.87 |
|  | 1.25 | 2.68 | 2.80 | 2.72 |  |  |  |  |  |
| **CD4+CTLA4+ cells/µL** | 15.79 | 37.64 | 35.99 | 39.70 |  | 0.05 | 0.05 | 0.21 | >0.99 |
|  | 30.88 | 44.65 | 35.52 | 56.66 |  |  |  |  |  |
| **CD8+CTLA4+ cells/µL** | 12.09 | 36.89 | 38.48 | 34.90 |  | 0.05 | 0.06 | 0.17 | 0.87 |
|  | 23.02 | 47.88 | 46.68 | 52.50 |  |  |  |  |  |
| **CD28+ (percent of CD4+)** | 98.16 | 96.43 | 95.47 | 97.22 |  | 0.13 | 0.15 | 0.23 | 0.88 |
|  | 1.89 | 4.97 | 6.04 | 2.05 |  |  |  |  |  |
| **CTLA4+ (percent of CD4+)** | 1.24 | 5.54 | 4.29 | 7.10 |  | <0.01 | 0.02 | 0.01 | 0.56 |
|  | 1.50 | 6.84 | 3.98 | 9.40 |  |  |  |  |  |
| **CD28+ (percent of CD8+)** | 77.22 | 79.63 | 82.87 | 75.59 |  | 0.40 | 0.09 | 0.63 | 0.12 |
|  | 7.59 | 9.31 | 7.38 | 10.33 |  |  |  |  |  |
| **CTLA4+ (percent of CD8+)** | 2.06 | 7.43 | 7.05 | 7.90 |  | 0.06 | 0.12 | 0.14 | 0.87 |
|  | 3.03 | 10.06 | 9.52 | 11.35 |  |  |  |  |  |
| **CD62L+CCR7+ Tcm (percent of CD4+CD45RO+)** | 58.77 | 54.94 | 56.33 | 52.95 |  | 0.42 | 0.51 | 0.52 | 0.71 |
|  | 8.96 | 12.79 | 4.78 | 19.85 |  |  |  |  |  |
| **CD62L-CCR7- Tem (percent of CD4+CD45RO+)** | 16.46 | 16.86 | 15.46 | 18.61 |  | 0.99 | >0.99 | 0.97 | 0.69 |
|  | 6.09 | 8.91 | 3.72 | 12.99 |  |  |  |  |  |
| **CD62L+CCR7+ Tcm (percent of CD8+CD45RO+)** | 21.33 | 17.77 | 17.49 | 18.12 |  | 0.10 | 0.15 | 0.23 | 0.87 |
|  | 6.98 | 9.10 | 5.02 | 12.99 |  |  |  |  |  |
| **CD62L-CCR7- Tem (percent of CD8+CD45RO+)** | 55.31 | 59.90 | 59.36 | 60.57 |  | 0.28 | 0.31 | 0.49 | >0.99 |
|  | 9.10 | 12.14 | 8.65 | 16.15 |  |  |  |  |  |
| **CD4+45RA+27+62L+ (percent of CD4+)** | 56.77 | 58.18 | 56.96 | 59.71 |  | 0.58 | 0.83 | 0.21 | 0.17 |
|  | 8.95 | 13.82 | 7.25 | 19.79 |  |  |  |  |  |
| **CD4+45RA-27+ (percent of CD4+)** | 32.46 | 28.98 | 31.11 | 26.31 |  | 0.24 | 0.64 | 0.12 | 0.17 |
|  | 8.38 | 7.09 | 4.92 | 8.74 |  |  |  |  |  |
| **CD8+45RA+27-62L- (percent of CD8+)** | 10.72 | 11.87 | 9.49 | 14.84 |  | 0.31 | 0.44 | 0.37 | 0.56 |
|  | 5.57 | 14.68 | 5.95 | 21.44 |  |  |  |  |  |
| **CD25+ (percent of CD4+)** | 35.38 | 33.47 | 37.46 | 27.09 |  | 0.15 | >0.99 | 0.04 | 0.02 |
|  | 10.7 | 8.59 | 7.76 | 5.78 |  |  |  |  |  |
| **CD25+ (percent of CD8+)** | 5.59 | 5.73 | 6.88 | 3.89 |  | 0.15 | 0.60 | 0.20 | 0.78 |
|  | 2.90 | 5.53 | 6.84 | 1.75 |  |  |  |  |  |
| **CD69+ (percent of CD4+)** | 5.48 | 8.00 | 8.48 | 7.15 |  | 0.04 | 0.06 | 0.22 | 0.74 |
|  | 2.08 | 4.04 | 4.80 | 2.36 |  |  |  |  |  |
| **CD69+ (percent of CD8+)** | 12.91 | 15.22 | 15.30 | 15.09 |  | 0.37 | 0.59 | 0.55 | 0.99 |
|  | 5.32 | 8.48 | 8.84 | 8.79 |  |  |  |  |  |
| **B cell Subsets** |  |  |  |  |  |  |  |  |  |
| **CD19^+^ cells/µL** | 350.30 | 336.80 | 335.10 | 338.90 |  | 0.66 | 0.73 | 0.74 | >0.99 |
|  | 165.40 | 142.60 | 139.50 | 156.20 |  |  |  |  |  |
| **CD19+ (percent of lymphocytes)** | 13.49 | 16.11 | 14.72 | 18.10 |  | 0.09 | 0.22 | 0.12 | 0.31 |
|  | 3.14 | 6.09 | 5.08 | 7.23 |  |  |  |  |  |
| **CD27+ total memory (percent of CD19+)** | 14.85 | 19.50 | 23.13 | 14.32 |  | 0.24 | 0.07 | 0.89 | 0.09 |
|  | 10.16 | 10.55 | 11.34 | 7.10 |  |  |  |  |  |
| **HLA-DR - Mean MFI of CD19+** | 2137.00 | 2083.00 | 2156.00 | 1992.00 |  | 0.95 | 0.84 | 0.70 | 0.74 |
|  | 707.20 | 713.50 | 709.30 | 756.50 |  |  |  |  |  |
| **CD21- (percent of CD19+) (immature)** | 2.36 | 3.49 | 2.89 | 4.23 |  | 0.08 | 0.28 | 0.07 | 0.37 |
|  | 1.17 | 2.36 | 1.16 | 3.26 |  |  |  |  |  |
| **CD21+ (percent of CD19+) (mature)** | 95.56 | 94.07 | 95.12 | 92.75 |  | 0.23 | 0.44 | 0.22 | 0.50 |
|  | 2.33 | 3.66 | 1.99 | 4.87 |  |  |  |  |  |
| **IgM+ (percent of CD19+)** | 48.08 | 49.30 | 44.21 | 55.40 |  | 0.96 | 0.85 | 0.80 | 0.53 |
|  | 22.89 | 19.75 | 26.02 | 6.62 |  |  |  |  |  |
| **CD24+IgM- (percent of CD19+) Plasmablasts** | 39.21 | 43.70 | 48.77 | 37.61 |  | 0.44 | 0.25 | >0.99 | 0.32 |
|  | 16.95 | 16.36 | 18.67 | 12.18 |  |  |  |  |  |
| **CD24+IgM+ (percent of CD19+) Transitional** | 47.82 | 44.96 | 41.01 | 49.72 |  | 0.68 | 0.52 | >0.99 | 0.53 |
|  | 20.76 | 16.09 | 19.83 | 10.18 |  |  |  |  |  |
| **CD27+IgD+IgM+ (percent of CD19+) nonswitched IgM memory** | 4.69 | 6.22 | 6.59 | 5.78 |  | 0.41 | 0.55 | 0.51 | >0.99 |
|  | 3.55 | 3.79 | 4.90 | 2.32 |  |  |  |  |  |
| **CD27+IgD-IgM+ (percent of CD19+) IgM-only memory** | 0.60 | 1.06 | 1.28 | 0.80 |  | 0.06 | 0.04 | 0.38 | 0.18 |
|  | 0.50 | 0.61 | 0.72 | 0.32 |  |  |  |  |  |
| **CD27+IgD-IgM- (percent of CD19+) class-switched IgM memory** | 4.79 | 10.64 | 13.12 | 7.66 |  | 0.03 | 0.02 | 0.28 | 0.32 |
|  | 4.16 | 7.13 | 8.37 | 4.38 |  |  |  |  |  |
| **NK cells** |  |  |  |  |  |  |  |  |  |
| **CD56^+^CD16^+^ cells/µL** | 173.10 | 163.60 | 127.10 | 209.10 |  | 0.33 | 0.15 | 0.99 | 0.37 |
|  | 84.88 | 119.50 | 44.82 | 166.80 |  |  |  |  |  |
| **CD56+16+ (percent of CD3+ lymphocytes)** | 3.90 | 5.85 | 4.13 | 3.68 |  | 0.66 | 0.50 | 0.47 | 0.74 |
|  | 2.88 | 4.71 | 5.04 | 4.14 |  |  |  |  |  |
| **Monocyte Subsets** |  |  |  |  |  |  |  |  |  |
| **CD14+HLA-DR^low/neg^ (percent of CD14+)** | 3.78 | 15.37 | 17.83 | 12.29 |  | 0.03 | <0.01 | 0.39 | 0.31 |
|  | 2.81 | 15.70 | 17.23 | 14.06 |  |  |  |  |  |
| **HLA-DR MFI of CD14+** | 637.70 | 346.30 | 355.90 | 386.40 |  | 0.09 | 0.14 | 0.45 | 0.85 |
|  | 501.40 | 197.40 | 208.30 | 205.70 |  |  |  |  |  |
| **Tie2 MFI of CD14+HLA-DR-** | 19.10 | 13.96 | 14.51 | 12.97 |  | 0.12 | 0.18 | 0.28 | 0.69 |
|  | 10.08 | 8.18 | 10.11 | 3.33 |  |  |  |  |  |
| **Tie2 MFI of CD14+HLA-DR+** | 18.31 | 35.59 | 45.77 | 17.27 |  | 0.30 | 0.12 | 0.84 | 0.23 |
|  | 5.44 | 32.46 | 37.10 | 4.34 |  |  |  |  |  |
| **CD14-16+ (percent of monocytes) Non-classical** | 8.10 | 4.90 | 4.44 | 5.40 |  | 0.22 | 0.23 | 0.44 | 0.69 |
|  | 6.60 | 2.70 | 1.90 | 3.51 |  |  |  |  |  |
| **CD14+16+ (percent of monocytes) Intermediate** | 20.30 | 24.90 | 26.76 | 22.55 |  | 0.61 | 0.65 | 0.73 | 0.87 |
|  | 8.89 | 15.90 | 18.74 | 12.23 |  |  |  |  |  |
| **CD14+16- (percent of monocytes) Classical** | 68.72 | 67.91 | 67.29 | 68.69 |  | 0.71 | 0.69 | 0.88 | 0.94 |
|  | 12.14 | 17.29 | 19.27 | 15.74 |  |  |  |  |  |
| **HLA-DR MFI of CD14-16+ monocytes** | 38.17 | 102.90 | 37.70 | 220.10 |  | 0.97 | 0.43 | 0.30 | 0.15 |
|  | 41.68 | 174.60 | 41.59 | 262.50 |  |  |  |  |  |
| **HLA-DR MFI of CD14+16+ monocytes** | 1148.00 | 991.10 | 967.90 | 1033.00 |  | 0.35 | 0.39 | 0.52 | 0.79 |
|  | 529.20 | 515.20 | 508.60 | 584.60 |  |  |  |  |  |
| **HLA-DR MFI of CD14+16- monocytes** | 544.80 | 285.80 | 283.60 | 289.80 |  | 0.05 | 0.08 | 0.20 | 0.74 |
|  | 449.00 | 157.40 | 155.10 | 180.00 |  |  |  |  |  |
| **Tie-2+ (percent of monocytes by FSC/SSC)** | 35.75 | 37.18 | 41.64 | 29.15 |  | 0.83 | 0.82 | 0.41 | 0.74 |
|  | 15.19 | 23.52 | 27.65 | 11.96 |  |  |  |  |  |
| **CD14-16+ (percent of Tie2+ monocytes)** | 21.42 | 22.42 | 22.20 | 22.81 |  | 0.86 | 0.85 | 0.56 | 0.79 |
|  | 18.37 | 17.96 | 20.09 | 15.51 |  |  |  |  |  |
| **CD14+16+ (percent of Tie2+ monocytes)** | 20.23 | 22.95 | 25.54 | 18.29 |  | 0.93 | 0.87 | 0.64 | 0.57 |
|  | 8.29 | 14.50 | 17.30 | 6.47 |  |  |  |  |  |
| **CD14+16- (percent of Tie2+ monocytes)** | 52.79 | 52.19 | 50.51 | 55.20 |  | 0.97 | 0.95 | 0.89 | 0.60 |
|  | 19.04 | 22.63 | 24.41 | 21.33 |  |  |  |  |  |
| **Tie2 MFI of CD14-16+ monocytes** | 29.45 | 37.86 | 39.63 | 34.68 |  | 0.03 | 0.03 | 0.30 | 0.34 |
|  | 9.20 | 10.13 | 11.01 | 8.45 |  |  |  |  |  |
| **Tie2 MFI of CD14+16+ monocytes** | 33.31 | 39.15 | 40.82 | 36.14 |  | 0.37 | 0.37 | 0.69 | 0.41 |
|  | 9.85 | 14.03 | 16.33 | 9.40 |  |  |  |  |  |
| **Tie2 MFI of CD14+16- monocytes** | 16.43 | 18.12 | 19.68 | 15.33 |  | 0.76 | 0.51 | 0.71 | 0.74 |
|  | 4.67 | 7.84 | 9.12 | 4.26 |  |  |  |  |  |
| **TNFRII MFI of monocytes by FSC/SSC** | 113.40 | 184.20 | 226.30 | 131.50 |  | 0.01 | <0.01 | 0.57 | 0.03 |
|  | 44.14 | 98.38 | 104.80 | 60.66 |  |  |  |  |  |
| **CD124 (IL4R-α) (percent of CD14+)** | 3.67 | 6.53 | 5.16 | 8.25 |  | 0.36 | 0.89 | 0.15 | 0.17 |
|  | 2.29 | 7.08 | 5.23 | 8.96 |  |  |  |  |  |
| **CD124 (IL4R-α) MFI of CD14+** | 8.99 | 9.90 | 10.19 | 9.54 |  | 0.07 | 0.05 | 0.34 | 0.35 |
|  | 1.36 | 1.48 | 1.67 | 1.20 |  |  |  |  |  |
| **CD40+ (percent of CD14+ cells)** | 38.31 | 37.29 | 42.15 | 28.55 |  | 0.97 | 0.64 | 0.41 | 0.18 |
|  | 20.92 | 20.64 | 21.96 | 16.48 |  |  |  |  |  |
| **CD80+ (percent of monocytes by FSC/SSC)** | 1.58 | 1.51 | 1.44 | 1.59 |  | 0.67 | 0.91 | 0.54 | 0.78 |
|  | 1.84 | 1.37 | 1.20 | 1.64 |  |  |  |  |  |
| **CD86+ (percent of monocytes by FSC/SSC)** | 99.59 | 99.21 | 99.11 | 99.34 |  | 0.95 | 0.80 | 0.48 | 0.74 |
|  | 0.31 | 1.28 | 1.53 | 0.97 |  |  |  |  |  |
| **B7H1 MFI of monocytes by FSC/SSC** | 19.18 | 17.08 | 17.21 | 13.47 |  | 0.78 | 0.64 | 0.44 | 0.20 |
|  | 5.62 | 7.43 | 5.17 | 7.18 |  |  |  |  |  |
| **CD142 MFI of CD14+** | 11.09 | 8.50 | 6.77 | 10.89 |  | 0.59 | 0.24 | 0.75 | 0.16 |
|  | 11.44 | 9.14 | 10.79 | 7.74 |  |  |  |  |  |
| **TLR4+ (percent of CD14+)** | 10.51 | 17.54 | 12.31 | 22.78 |  | 0.19 | 0.40 | 0.21 | 0.49 |
|  | 8.52 | 16.64 | 5.98 | 22.25 |  |  |  |  |  |
| **Other** |  |  |  |  |  |  |  |  |  |
| **CD64 MFI of Granulocytes by FSC/SSC** | 22.45 | 23.35 | 26.40 | 19.54 |  | 0.81 | 0.56 | 0.25 | 0.23 |
|  | 6.40 | 10.95 | 13.24 | 6.02 |  |  |  |  |  |
| **CD66b MFI of Granulocytes by FSC/SSC** | 228.50 | 234.20 | 235.20 | 233.10 |  | 0.42 | 0.55 | 0.49 | 0.81 |
|  | 120.30 | 76.49 | 89.03 | 63.38 |  |  |  |  |  |
| **CD15+14- MDSCs (percent of PBMNCs)** | 1.04 | 0.78 | 0.55 | 1.07 |  | 0.02 | 0.02 | 0.12 | 0.81 |
|  | 0.70 | 1.08 | 0.22 | 1.60 |  |  |  |  |  |
| **CD66b MFI of CD15+14- PBMNCs** | 88.74 | 100.10 | 85.64 | 109.20 |  | 0.69 | 0.90 | 0.57 | 0.52 |
|  | 100.90 | 117.90 | 113.20 | 118.60 |  |  |  |  |  |
| **Lin-DR+ DCs (percent of PBMNCs)** | 1.91 | 2.00 | 1.71 | 2.36 |  | 0.83 | 0.41 | 0.65 | 0.95 |
|  | 0.72 | 1.08 | 0.25 | 1.57 |  |  |  |  |  |
| **Lin-DR-CD33+ MDSCs (percent of PBMNCs)** | 1.73 | 1.56 | 1.34 | 1.85 |  | 0.75 | 0.37 | 0.64 | 0.24 |
|  | 0.86 | 0.72 | 0.75 | 0.61 |  |  |  |  |  |
| **Lin-DR-CD33+ MDSCs (percent of total cells)** | 0.84 | 0.76 | 0.63 | 0.92 |  | 0.22 | 0.06 | >0.99 | 0.09 |
|  | 0.36 | 0.51 | 0.55 | 0.45 |  |  |  |  |  |
